# Chronic postoperative pain after non-intubated uniportal VATS lobectomy

**DOI:** 10.3389/fsurg.2023.1282937

**Published:** 2023-11-13

**Authors:** Attila Farkas, Tímea Csókási, Csongor Fabó, Zsolt Szabó, Judit Lantos, Balázs Pécsy, György Lázár, Ferenc Rárosi, László Kecskés, József Furák

**Affiliations:** ^1^ Department of Thoracic Surgery, Markusovszky University Teaching Hospital, Szombathely, Hungary; ^2^ Department of Pulmonology, University of Szeged, Szeged, Hungary; ^3^ Department of Anesthesiology and Intensive Therapy, University of Szeged, Szeged, Hungary; ^4^ Institute of Surgical Research, University of Szeged, Szeged, Hungary; ^5^ Department of Neurology, Bács-Kiskun County Hospital, Kecskemét, Hungary; ^6^ Department of Surgery, University of Szeged, Szeged, Hungary; ^7^ Department of Medical Physics and Informatics, University of Szeged, Szeged, Hungary

**Keywords:** post-thoracotomy pain syndrome, chronic pain, intubated, non-intubated, uniportal, VATS

## Abstract

**Introduction:**

Patients undergoing thoracic surgery are at increased risk of developing, long-lasting pain. Beyond the non-surgical factors, the type of operation, including the number of incisions, and the anesthetic assessment seemed to be important factors, although some studies are controversial. The aim of our study was to examine the presence of chronic postoperative pain after non-intubated uniportal VATS lobectomy. We examined the difference between the intubated, relaxed and non-intubated spontaneous ventilation surgical approaches in patients who underwent video-assisted thoracoscopic (VATS) uniportal lobectomy.

**Methods:**

Demographic and postoperative data were retrospectively collected and analyzed, focusing on the use of pain medications, in 67 patients of the 140 patients selected by propensity score matching who underwent intubated (iVATS) or non-intubated (NITS) uniportal VATS lobectomy. This study focused on the use of analgesic medications 3, 6, and 12 months after surgery.

**Results:**

Thirty-five intubated and 32 non-intubated patients were compared. Although the analgesic consumption was nearly 2% higher among the iVATS patients during the follow-up period, there were no statistically significant differences at 3 months (15.6 vs. 17.1%) (*p* = 0.868), at 6 months (9.4 vs. 12.4%) (*p* = 0.785), and at 12 months (3.3 vs. 5.9%) (*p* = 0.633) between the NITS and iVATS groups, respectively. More female than male patients reported chronic pain, but the difference was not statistically significant (*p* = 0.616). Diabetes mellitus was a statistically significant cofactor associated with chronic pain (*p* = 0.03), while cardiac disease (*p* = 0.6), perioperative morbidity (*p* = 0.228), prolonged air leak (*p* = 0.057), and repeat drainage (*p* = 0.626) were not.

**Conclusion:**

Our study suggests that after non-intubation VATS lobectomies, the postoperative pain was less at 3, 6, and 12 months in NITS patients compared to iVATS patients. The 2% difference was not significant, so it may not be appropriate to claim the advantages of NITS in terms of postoperative pain.

## 1. Introduction

Thoracic surgical incisions, including thoracotomy and video-assisted thoracic surgery (VATS) incisions, pose an increased risk of chronic pain postoperatively. The point prevalence is nearly 25%, and one-third of these patients have a neuropathic component (1). Approximately 50% of patients with persistent pain eventually develop some limitations in their daily life activities, and nearly 30% of them have sleeping disturbances (2). Its incidence decreases over time, but late onset also occurs (3). Apart from the thoracic surgical incision, multiple risk factors may contribute to facilitate the onset of chronic pain after thoracic procedures. The most important factors are the American Society of Anesthesiologists physical status classification, age below 60 years, female sex, preoperative hypertension, postoperative non-patient controlled analgesia regimen for pain management, long-term chest tube drainage after the surgery (more than 4 days), number of chest drains, and postoperative chemoradiotherapy (1, 3–5). The association between acute postoperative pain and persistent pain is debatable. Some studies have shown that the intensity of pain soon after the operation (from 1 day to 1 week postoperatively) is predictive of chronic pain (4, 6–11), whereas others have found no correlation (5, 12). Approximately 50% of patients undergoing “traditional” thoracotomies suffer from chronic pain 3–6 months after the surgery (13). Injury to the intercostal nerve during the thoracotomy is considered the primary source of pain. Skin incision, rib spreading or resection, muscle splitting, costovertebral joint disruption, and chest tube or surgical drain insertion can contribute to this process (14). Additionally, the placement of pericostal sutures or wires at closure can potentially harm the intercostal nerve (15).

In the last few decades, VATS has shown unquestionable surgical advantages over open thoracotomy, including early removal of chest tube, faster functional recovery, a shorter hospital stay (16) and improved pulmonary outcomes (17). Furthermore, the 5-year overall survival for early-stage lung cancer was better in patients after VATS lobectomy compared to those who underwent open thoracotomy (18). Additionally, post-surgical pain and functional impairment appear to be reduced (19). In the last few decades, aside from the original multiportal VATS, other modified thoracoscopic approaches have been presented and spread worldwide. Currently, one of the most popular modifications from the surgeon's perspective is the uniportal VATS approach (UniVATS), wherein the camera and all instruments are placed in a single utility incision (20). With regard to anesthetic management, although the “gold standard” for VATS has been the intubated general anesthesia (iVATS) approach, other possibilities are now available. In 2004, Pompeo et al. published the initial experiments on non-intubated video-assisted thoracoscopic surgery (NITS) for solitary pulmonary nodule resection (21). Since then, this technique has become increasingly accepted, and its advantages and disadvantages have become more apparent. Early hospital discharge and mortality are lower in non-intubated patients compared to patients who underwent tracheal intubation (22). Intubation-associated discomfort and complications, such as tracheal damage, vocal fold paralysis, respiratory function impairment, and alveolar barotrauma, can be avoided. General anesthesia increases the risk of intraoperative hypoxia, and ventilator dependence after the surgery, with potentially multiple organ failure, septic infections, and intensive care unit induced neuromyopathy or polyneuropathy (23). All these features originate from the difference in anesthetic management because the thoracic surgery technique is the same in NITS and conventional iVATS.

In our study, we compared the presence of persistent pain up to 12 months after surgery as defined by differences in analgesic consumption in patients who underwent lobectomy using the iVATS and NITS techniques.

## 2. Methods

### 2.1. Patients

Using propensity score matching, we selected 70 iVATS and 70 NITS patients who underwent surgery between July 3, 2015, and November 27, 2018, in the Department of Thoracic Surgery at the Szeged University Hospital. The following factors were used for propensity score match: age, sex, BMI, Charlson Comorbidity Index, histology, type pf lobectomy and FEV1. We compared the differences between two groups with Mann-Whitney *U* test. From these patients, 35 iVATS and 32 NITS patients had complete documentation of postoperative pain and use of analgesic medications, and only their data were used in this study. All patients underwent the same preoperative pulmonary examinations and none of them had chronic chest pain before the surgery. All patients who were selected in this study had BMI less than 30 or close to it, in both groups. Between July 02, 2015, (at the beginning of the uniportal iVATS procedure) and January, 24, 2017 (at the beginning of the uniportal NITS VATS procedure) all the patients underwent iVATS. The NITS was started at January, 24, 2017. After the start of the NITS till the end of the study (November, 27, 2018), if the anesthesiologist in charge was familiar with the NITS procedure, the lung resection was performed with NITS, but in the other cases the traditional iVATS was our choice. Age-adjusted Charlson Comorbidity Index was calculated with regard to comorbidities and weighted it based on patient age. The exclusion criteria were similar: tumors greater than 7 cm, cN2 lymph node stage, previous thoracotomy, therapeutic –dose anticoagulation, unstable cardiac disease, psychiatric disorders, or severe obesity with 35 <body mass index (BMI). The patients who underwent conversion from NITS to IVATS or from VATS to open method were excluded from this study. Additionally, the possibility of difficult intubation was excluded in cases of NITS.

### 2.2. Surgical procedures

Although the anesthetic considerations were different, the number of surgical incisions was the same in both groups: All patients were operated via uniportal technique, and an utility incision was performed in the 5th intercostal space without any additional port. All the skin incisions were less than or equal to 4 cm. Local anesthesia with nerve blockade is crucial in NITS. Before the incision, the skin was infiltrated with local anesthetic (2% lidocaine, 5 mg/kg) between the 5th and 6th rib on the middle axillary line. After the utility incision was completed, vagus nerve blockade was performed with 0,5% bupivacaine (0.5 ml/kg) on the right upper mediastinum or on the aortopulmonary window. The paravertebral blockade was administered with 4–5 ml of bupivacaine alongside the thoracic spine, blocking the 2nd to the 5th intercostal nerves.

Subsequently, the surgical steps were similar; however, the non-intubated patients were able to breathe spontaneously under the procedure. All the patients underwent uniportal VATS lobectomy and radical lymph node dissection. Finally, a plastic chest tube was placed through the utility incision, and the wound was closed. None of the patients underwent intraoperative conversion from video-assisted thoracoscopy to open thoracic surgery or from NITS to iVATS.

The duration of paravertebral blockade was 24 h. After that we used intravenous Neodolpasse (diclofenac) and Paracetamol infusion on the first postoperative day to reduce acute postoperative pain. On the next day we switched to oral diclofenac therapy.

### 2.3. Data collection

The Human Investigation Review Board of University Szeged approved and reviewed the study. All patients were informed and gave written consent. All patients were assessed during follow-up visits in our pulmonology department at 3, 6, and 12 months after the surgery. General conditions, pain status, type of pain medication, analgesic consumption, and current wound healing status were recorded. In our study the severity of acute pain and and the related painkiller consumption in the early postoperative period were not recorded. Chronic pain was defined as pain that develops or increases in intensity after a surgical procedure and persists beyond the healing process for at least 3 months after the surgery, according to the International Association for the Study of Pain (IASP. Pain is localized closely to the incision, but can spread to other part of the chest. Although intensity of pain is variable, neuropathic component and sensory disturbances of the wound can be observed (24).

## 3. Results

Age, sex, BMI, Charlson Comorbidity Index, history of cardiac disease, diabetes mellitus, forced expiratory volume in 1 s (FEV1) or diffusing lung capacity for carbon monoxide (DLCO) were not significantly different in patients who underwent intubated and non-intubated VATS lobectomies. Patient demographics and preoperative pulmonary evaluation are presented in Table 1.

**Table 1:** Patient demographics.

	NITS (*n* = 32)	iVATS (*n* = 35)	*p*-value
Numbers based on gender (%)			
Female	22 (68.75%)	23 (65.72%)	
Male	10 (31.25%)	12 (34.28%)	
Mean age	64.4 (52–78)	65.9 (56–80)	0.746
BMI (kg/m^2^)	25.48 (18.5–32.8)	25.63 (19.5–37.2)	0.724
FEV1 (%, mean)	92.6% (42–125)	89.5% (48–144)	0.014
DLCO (%, mean)	72.73% (40–96.4)	73.66% (35–126)	0.525
Diabetes mellitus no. (%)	6 (18.75%)	4 (11.42%)	0.226
History of cardiac disease no. (%)	8 (25%)	6 (17.14%)	0.682
Charlson Comorbidity Index point	4.78 (2–9)	4.88 (2–9)	0.123

* BMI, body mass index; FEV1, forced expiratory volume in 1 s; DLCO, diffusing lung capacity for carbon monoxide.

The *p*-values showed no statistically significant differences in the mean operation time, mean day of chest tube removal, prolonged air leak, number of repeat drainages, reoperation, or morbidity. The mean total operation time among the patients with chronic pain was 100 min (85–125), which was 7 min longer as in the total cohort. This difference was not significant. No perioperative mortality was observed in the follow-up period. Pathological types and stages showed also not significant differences between the groups. Surgical data, pathological types, and staging are listed in Table 2.

**Table 2:** Surgical and pathological data.

	NITS (*n* = 32)	iVATS (*n* = 35)	*p*-value
Mean total operation time (min)	94.2 (60–175)	93.22 (45–160)	0.215
Prolonged air leak no. (%)	1 (3.1%)	5 (14.2%)	0.465
Repeat drainage no. (%)	0 (0%)	2 (5.6%)	0.173
Mean day of chest tube removal	2.4 (1–7)	4.2 (1–18)	0.602
Morbidity no. (%)	1 (3.1%)	9 (25.7%)	0.597
Return to operating room no. (%)	0 (0%)	1 (2.8%)	0.339
Mortality no. (%)	0 (0%)	0 (0%)	
Pathological types no. (%)			0.351
Adenocarcinoma	24 (75%)	24 (68.5%)	
Squamous cell carcinoma	5 (15.6%)	5 (14.3%)	
Other	3 (9.4%)	6 (17.2%)	
Pathological staging no. (%)			0.984
I A	19 (59.4%)	19 (54.3%)	
I B	6 (18.8%)	4 (11.4%)	
II A	2 (6.2%)	3 (8.6%)	
II B	3 (9.4%)	3 (8.6%)	
III A	2 (6.2%)	5 (14.3%)	
III B	0 (0%)	0 (0%)	
IVA	0 (0%)	1 (2.8%)	

With female predominance, although statistically not significant (*p*-value = 0.616), both sexes reported chronic pain during the initial 2 follow-up periods (16.7% vs. 16% at 3 months and 11.9% vs. 8% at 6 months). Surprisingly, after 1 year, no men reported the presence of chronic pain (7.9% vs. 0%).

Patients with prolonged air leaks were more likely to develop persistent pain in comparison to patients whose chest tubes were removed within 5 days (33.3% vs. 14.8% at 3 months, 33.3% vs. 8.2% at 6 months, and 16.7% vs. 3.4% at 12 months).

Although statistically not significant (*p*-value = 0.228), perioperative morbidity also appeared to be a risk factor in the first 2 follow-up periods (30% vs. 14% at 3 months, 20% vs. 8.7% at 6 months), but after 12 months, no patient with morbidity reported any chronic pain (0% vs. 5.2%).

Only one patient required reoperation, and this patient suffered from chronic pain during follow-up (*p*-value = 0.003). Interestingly, none of the two patients who underwent repeat drainage reported chronic analgesic consumption (*p*-value = 0.626).

Patients with diabetes mellitus had a higher chance of long lasting pain than non-diabetics (30% vs. 14% at 3 months, 30% vs. 7% at 6 months, 10% vs. 3.5% at 12 months), and this difference was statistically significant (*p*-value = 0.03). In terms of cardiac disease, the results were similar (28.5% vs. 13.2% at 3 months, 14.2% vs. 9.4% at 6 months, and 14.2% vs. 1.8% at 12 months), but not significant (*p*-value = 0.6). Table 3 shows the relationship between the presence of chronic pain and various demographic and surgical factors.

**Table 3:** Association between the presence of chronic pain and various demographic and surgical factors.

	Presence of chronic pain (%)			*p*-value
	3 months	6 months	12 months	
Female (*N* = 45)	16.7%	11.9%	7.9%	0.616
Male (*N* = 22)	16%	8%	0%	
Prolonged air leak (*N* = 6)	33.3%	33.3%	16.7%	0.057
No prolonged air leak (*N* = 61)	14.8%	8.2%	3.4%	
Perioperative morbidity (*N* = 10)	30%	20%	0%	0.228
No perioperative morbidity (*N* = 57)	14%	8.7%	5.2%	
Reoperation (*N* = 1)	100%	100%	100%	0.003
No reoperation (*N* = 66)	15%	9%	4.5%	
Repeat drainage (*N* = 2)	0%	0%	0%	0.626
No repeat drainage (*N* = 65)	16.4%	10.4%	4.4%	
Diabetes mellitus (*N* = 10)	30%	30%	10%	0.03
No diabetes mellitus (*N* = 57)	14%	7%	3.5%	
Cardiac disease (*N* = 14)	28.5%	14.2%	14.2%	0.6
No cardiac disease (*N* = 53)	13.2%	9.4%	1.8%	

During follow-up at 3 months, 15.6% of NITS and 17.1% of iVATS patients reported the use of pain medications. These data showed a 1.5% difference between the two groups, which was not statistically significant (*p*-value = 0.868). After 6 months, both rates were reduced, with 9.4% of NITS and 11.4% of iVATS patients needing regular pain medications. The difference was 2%, which was again not significant (*p*-value = 0.785). There was a further reduction in pain at 12 months, with only 3.3% of NITS and 5.9% of iVATS patients reporting analgesic use. The difference was 2.6% but was again not significant (*p*-value = 0.633).

In the NITS group, the ratio of patients using pain medications decreased from 15.6% to 3.3% in 12 months. The NITS patients who reported chronic pain at 3 months, 60% continued to use pain medications at 6 months, further decreasing to 20% at 12 months.

In the iVATS group, the rate of patient-reported analgesic consumption also declined from 17.1% to 5.9% in 1 year. Of iVATS patients who used pain medications at 3 months, 66.6% reported continued use at 6 months, further decreasing to 33% at 12 months.

The relationship between the presence of long lasting pain and the type of surgery performed is seen in Table 4.

**Table 4:** Association between the presence of chronic pain and the type of surgery performed.

	NITS (*n* = 32)	iVATS (*n* = 35)	*p*-value
Presence of chronic pain no, (%)			
3 months	5 (15.6%)	6 (17.1%)	0.868
6 months	3 (9.4%)	4 (11.4%)	0.785
12 months	1 (3.3%)	2 (5.9%)	0.633

## 4. Discussion

The pathophysiology of healing and regeneration is complex, and many steps remain unclear. The neuroinflammatory system, which regulates pro- and anti-inflammatory cytokines, promotes the restoration of normal (painless) function (25). Therefore, disturbed activation of the immune and inflammatory systems is crucial in the appearance and progress of chronic pain.

The secretion of neuroinflammatory mediators facilitates neuroimmune activation, sensitizes primary afferent neurons, and contributes to the various symptoms of neuralgia (26). The activation in proinflammatory cytokines plays a key role in neuropathic pain. The induction of tumor necrosis factor (TNF), interleukin-1 (IL-1), and interleukin-6 (IL-6) in the spinal cord and the dorsal root ganglion, contributes to chronic hyperalgesia and allodynia (27). Nerve growth factor (NGF) has a cytokine-like action on mast cells, basophils, neutrophils, and lymphocytes and is strongly associated with hypersensitivity (28). Decreased expression in immunosuppressive cytokines like interleukin-4 (IL-4) or interleukin-10 (IL-10) is also significant between pain and painless neuropathy (29). Similarly, the reduction of transforming growth factor-ß1 (TGF-ß1) is also significant in skin cell fibromyalgia syndrome (30).

The interruption of tissue continuity indicates an increase in inflammatory mediator level. Prostaglandin, histamine, and bradykinin can stimulate the nociceptors and lead to an increased activation and reduction in pain threshold. This primary sensitization leads to acute postoperative pain. The permanent activation of sensory receptors after the surgery provokes excessive sensibility of dorsal horn neurons and the central nervous system amplifies its response. Finally, the increased neural reactivity lead to central sensitization and with neuropathic component can transform to persistent, chronic pain (31).

VATS is considered a less invasive and traumatic approach because of the smaller incision, which prevents rib spreading and resection. Many studies have confirmed that thoracotomy is related with a higher possibility of clinically relevant constant pain and pain-connected limitations and dysfunctions in normal daily life activities (mean follow-up time, 3–36 months) compared to VATS (19, 32–36). These findings are consistent with reduced effects on systemic immune responses and cellular immune functions (37). VATS reduces circulating CD3^+^, CD4^+^, and CD8^+^ T cells (38, 39) while thoracotomy results in significantly stronger immune suppression of NK cells and T lymphocytes (40). Less T-cell suppression decreases the risk of imbalanced immune regulation, tumor growth, and recurrence. IL-6 is significantly more elevated in patients who undergo thoracotomy than in those who undergo VATS, which may contribute to the lower incidence of chronic pain after minimally invasive surgery (41).

In contrast, other studies have shown no differences between the VATS and thoracotomy approaches (mean follow-up time, 3–33.5 months) in the onset and intensity of chronic pain after the surgery (42–45).

Theoretically, a smaller incision reduces immune activation and pain. There are slight differences in the postoperative systemic inflammatory response in UniVATS compared to multiportal VATS (46), but the presence of acute (47) and chronic pain is less frequent after the uniportal approach (11, 48, 49). A systematic review and meta-analysis showed slightly better postoperative results for UniVATS lobectomy than for the traditional multi-portal approach in the treatment of lung cancer (50).

Immune activation appeared to be affected by the type of anesthesia used as well. NITS has a lower negative effect on the activation of the cellular and humoral immune systems, which tend to be important in postoperative infections, pain, and tumor progression (51–53).

In our study the inflammatory and anti-inflammatory markers of the patients were not measured. We based our study on previous studies in connection with inflammatory response. Our initial hypothesis was that the non-intubated surgical approach reduces immune activation and causes a milder immune response, consequently lowering the presence of chronic pain in comparison to that in patients subjected to general anesthesia. This hypothesis has been confirmed in association with acute postoperative pain. Apart from the number of incisions, the intensity of pain is low in patients who undergo thoracic surgery without intubation (54–56). Pompeo et al. confirmed that the visual analog scale (VAS) score in 24 h with non-intubated 3-port access is lower than that of the intubated approach when performing wedge resection of solitary pulmonary nodules (21). The same result was found in patients with malignant pleural effusions who underwent talc pleurodesis using a single flexible trocar (57). Hwang et al. showed differences in the VAS scores in the first hour after uniportal bullectomy under sedation for primary spontaneous pneumothorax; however, this was not significant after 24 h (58). Zang et al. conducted a meta-analysis based on 14 randomized controlled trials and found that the VAS score was significantly milder in patients who were not intubated (59). According to Wei et al., acute pain was also milder in children aged 3–8 years who underwent VATS (60).

Some authors have drawn different conclusions; Kocatürk et al. found no significant difference at 4, 8, 12, and 24 h between awake and intubated patients who underwent diagnostic operations for pleural disease (61). Yang et al. followed non-intubated and intubated patients after uniportal VATS lobectomy and showed that there was no difference between acute and chronic pain 3 months (52). In the literature, this study is the only one to compare chronic pain (within 3 months) between patients who underwent intubated and non-intubated uniportal lobectomies. In other studies that examined acute pain after surgery, the duration of the interventions was relatively short compared to that of lobectomy. Longer operations may activate the immune response more and maintain the activation at a higher level for longer durations.

To our knowledge, our study is the first to examine the differences in chronic pain between patients who underwent intubated and non-intubated lobectomy with a follow-up period of 1 year. We evaluated the presence of chronic pain based on analgesic consumption at 3, 6, and 12 months after surgery. No pain scales were used. We hypothesized that patients who continue to use pain medications months after surgery experience significant pain levels, causing limitations in daily life activities. All patients underwent single-incision VATS lobectomy, and there were no statistically relevant differences in the demographic, pulmonary, surgical, or pathological parameters. The main difference between the 2 groups was the anesthetic management; therefore, theoretically, all the resulting differences in connection with long-lasting pain originate from the anesthetic assessment and the type of ventilation (mechanical single-lung ventilation vs. spontaneous ventilation) used. Based on our results, there was no significant difference between the two groups, although a higher number of the iVATS patients needed pain medications at home after surgery (1.5% more at 3 months, 2% more at 6 months, and 2.6% more at 12 months). This finding is similar to that of Yang et al., but our follow-up time was 9 months longer. In addition, we found no statistically significant differences in the onset of chronic pain between female and male patients; however, in our cohort, more female patients had long-lasting symptoms. Furthermore, none of the male patients reported analgesic use 12 months after surgery. Neuropathy and neuropathic pain are one of the most common complication of diabetes. The pathophysiology is not clear put hyperglycemia, oxidative stress, metabolic and microvascular changes seem to be important (62). The statistically significant higher incidence of the chronic pain among our diabetic patients suggests that diabetes may contribute to neuropathic component of chronic pain. Although diabetes mellitus was the only factor that was statistically significant, perioperative morbidity and the presence of cardiac disease also seemed to contribute to a higher risk of chronic pain, similar to the findings of many previous studies (63–65). A higher percentage of patients with prolonged air leaks experienced chronic pain; however, the need for repeat drainage was not a risk factor. Retrospective studies, such as that by Peng et al., found a significantly higher incidence of chronic pain in patients with prolonged air leakage (1), but this connection seemed less clear in other reports (5, 66). Only one patient required reoperation in our study, and he reported chronic pain throughout the entire follow-up period. Because of the small number, the statistical significance was difficult to assess. In summary, less postoperative pain results in increased quality of life, faster recovery, an earlier return to work, and reduced analgesic consumption. Previous studies reported that the severity of acute pain is milder in patients undergoing NITS procedures compared to iVATS procedures after the surgery. Although the results of our study were not statistically significant, the onset of chronic pain was less frequent in NITS patients compared to iVATS patients. The analgesic consumption did not show a statistically significant difference between the two groups, even though it was nearly 2% higher among the iVATS patients during the follow-up period. Therefore, it may not be appropriate to claim the advantages of NITS in terms of postoperative pain, but the NITS had no disadvantages in terms of chronic pain. However, as every step of the pathophysiology has not yet been identified, further examination is needed to clarify this question.

## Data Availability

The original contributions presented in the study are included in the article/Supplementary Material, further inquiries can be directed to the corresponding author.
